# The monk who wants to sell his zen…

**DOI:** 10.4103/0972-0707.44045

**Published:** 2008

**Authors:** Velayutham Gopikrishna

**Affiliations:** Editor-In-Chief, Journal of Conservative Dentistry, Department of Conservative Dentistry, Meenakshi Ammal Dental College, Alapakkam Main Road Maduravoyal, Chennai - 600 095, Tamilnadu, INDIA Email: editor@jcd.org.in

I indeed consider this a great honour and privilege to head the editorial board of the Journal of Conservative Dentistry. I feel humbled to have eminent people like Padmabhushan Dr. Anil Kohli, Dr. A. P. Tikku, Dr. K. S. Banga, and the FODI for having bestowed their faith in me and for having chosen me for this endeavour. I also thank Dr. Lakshmi Narayanan and Dr. Kandasamy for their constant moral support and guidance. We shall work as a team to change the face of our speciality and scale newer heights.

## DISCLAIMER

The title of this article is a feeble attempt to mimic the phenomenal success attained by Robin Sharma's *The Monk who sold his Ferrari*!!!

I profess to be neither a picture perfect person to be emulated nor a philosophical person knowing the mantra for true personality development. I am just another ‘somebody’ like most people. Life teaches us a lot of lessons and one of the lessons that we learn the hard way is:

‘Experience is the comb that life gives you once you become bald!’

‘Experience is the name everyone gives to their mistakes!’

This pertinent fact has been known from time immemorial; yet we all try to learn from our own mistakes, while it is far wiser to learn from the mistakes of others.

Right now I am facing a peculiar dilemma, as I can neither qualify to be a beginner in the journey of life nor have I become completely bald and worldly wise yet! Hence, I am safely taking refuge in the experiences of great men and women whose values and principles are worth emulating.

As we look back to our student days with nostalgia, we do have a sense of pride and guilt. Pride for what we did and guilt for all that we could not achieve. So, in our eternal quest for SUCCESS, a metaphor, which varies from person to person, we lose track of our bearings and try to blame our shortcomings and inefficiency on things called fate, luck and circumstances.

The most salient truth for changing one's life and character is that any CHANGE should be within oneself. One should not try to change the situations and challenges that we face. This article is a frozen thought of all the ingredients that go into moulding a character, which in turn can mould one's destiny.

In my five years of undergraduate study at the Annamalai University, life in college and the hostel taught me many lessons. One of my observations was the lack of self-confidence in me and amongst many of my peers. The one common factor in all my seniors who were achievers was their self confidence. Hence, the first lesson in life is to like and believe in yourself and your abilities. If you do not have confidence in yourself, who else will?

## NEVER REJECT YOURSELF

More often than not, we don't get what we desire, not because of others but because we reject ourselves. Every time we experience fear of rejection, we should realize that it's not the person who is being rejected, but the action. Rejections are not personal rejections. Rejections only indicate that we have to correct or change our approach. By rejecting ourselves, we are depriving ourselves of getting what we want. Don't reject yourself anymore. We should realize that it isn't our business to be bothered about whether we will be accepted or rejected; it is the world's business. Our business is to put our best foot forward and extend our hand… Yes, there is a 50% possibility that our hand will be pushed away… but also a 50% possibility that we will get a handshake in return!

## BE ORIGINAL

As a frightful beginner to clinical practice after completing my bachelor's degree, I felt scared to face the world. I blamed this lack of self-belief on my not being a Master of anything! Hence, I joined my MDS, in the belief that it would rectify my ineptitude. However, after graduating I again had the same sinking feeling in my stomach, and this time had no excuse to offer. The common refuge for most of us who face such a situation is to try and copy or emulate others. This ‘herd philosophy’ of blindly trying to follow others is a recipe for disaster. This concept is of paramount importance when one tries to establish clinical practice. I have seen that most of us try to ‘survey’ the place where we want to establish and commonly follow some principles:

- We try to look for a place where there are no other clinics!
- We try to price our services at a lesser premium than others!

To be frank, even I followed these principles… but I have realized that a successful practitioner is one who cannot be easily COPIED! If you look around, you will agree with me that the law of human nature is: whatever can be copied will be copied. The corollary to this law is: that which cannot be copied will never be copied. From a clinician's point of view, it is foolish to compete only on the basis of selling price, promises and others' lack of knowledge. All these may give a temporary edge, short-time profits and turnover. More importantly, all of these may be easily copied.

As a consultant visiting a number of clinics, I have realized that clinics that do well are those that cannot be copied easily! The fundamental principles on which these successful practitioners operate are

- Quality treatment with a ‘no compromise’ attitude
- Personal rapport with patients (This should not be mistaken for smooth talking!)
- Great service with genuine goodwill
- Understanding the needs of the patient
- Trust and dependability

Cost of treatment and location of practice do not Figure in the above list! If you look around you will find that there are people who prefer Jet Airways, although it is more expensive than many other airlines; some people will travel an extra mile to go to their favourite food joints, although there might be cheaper joints nearby! The bottom line is that the key to a successful practice is … YOU! If you want your life and practice to be one of a kind, then make changes in yourself to become UNIQUE.

## FEEL LUCKY

I still remember the days in hostel, when we made a list of the so called ‘Lucky Guys’: SS for having a girl friend, SC for having a very rich dad, PP for being lucky in exams and so on. Now, over time, I have deciphered this magic of LUCK. In my understanding, this phenomenon of luck, on which I either blame my failure or credit everyone else's success, is nothing but my inability to understand the originality and uniqueness embedded in every creation. No two leaves are the same. Then how can two lives be the same? Irrespective of my best efforts, trying to be someone other than what I can be can never lead to success or prosperity. We can go on blaming luck for what we are or what we are not. The reality remains that each one of us will have a different set of opportunities. In my understanding, it is not luck but the drive to search for the best in ourselves that leads to success. Do not wait to become lucky. Create your own luck.

## CREATE TIME

In the past few years of leading a life of an academician and consultant, one of my common remarks used to be, ‘I am busy and have no time at all.’ Initially, this used to give me a thrill, as I mistook this to be the path to success. Now I realize that when my daughter wants me to read her a bedtime story, I do not have TIME. I have realized that lack of time means lack of control on your own life!

Time management isn't clock management. It is our ability to say ‘yes’ to what is important, and more importantly, the discipline to say ‘no’ to the ‘you don't have to do’ aspects of life. I have realized that my time is being stolen by the ‘trivial many’ that have little or no consequences on the overall effectiveness of my life. Time management is nothing but the art of compiling a ‘Not to do list’. A strict ‘no - no’ to the trivial things in one's life. These may vary from person to person; it could be ritualistic television watching, oversleeping or simply reading every bit of news in the papers, from obituaries to tenders!

Unused money devalues. Unused talent diminishes. Unused potential disintegrates. Unused time dies. Unused knowledge becomes a burden. What isn't used is abused. The tragedy of life isn't the ultimate death, but the resources that die within you when you are still alive. Use it, or you will lose it.

I would like to conclude with a few timeless quotes that continue to enlighten and inspire us:

- The difference between stumbling blocks and stepping-stones is in how you use them.
- I'm a great believer in luck, and I find that harder I work, the more I have of it!
- In the fight between the Rock and the Stream, the rock always loses!
- You drown not by falling into a river, but by staying submerged in it!

## MY MOTTO

If fate leaves me with a lemon, I will make lemonade out of it!

May you and this world discover you at the earliest!

